# CO-DESIGNING SOLUTIONS WITH WORKERS: SETTING RESEARCH AND POLICY PRIORITIES

**DOI:** 10.1093/geroni/igae098.1988

**Published:** 2024-12-31

**Authors:** Annie Rhodes, Leland Waters

**Affiliations:** Virginia Commonwealth University, Richmond, Virginia, United States; Virginia Commonwealth University, Richmond, Virginia, United States

## Abstract

An emerging body of literature has linked the quality of care for care recipients with the quality of work experience with the professional caregiver across the spectrum of long-term services and supports. For instance, prior to the COVID-19 vaccine, nursing home workers who worked in multiple locations were significantly more likely to be infected with COVID-19 and, subsequently, more likely to infect care recipients. Despite the natural alignment with worker and recipient interests, LTSS quality improvement efforts often do not focus on aligning recipient and worker perspectives as a reform and quality improvement strategy. For instance, although current evidence supports that staffing ratios improve both recipient care and caregiver safety, there has been a backlash against a proposed staffing mandate in nursing homes by provider stakeholders. To uplift the caregiver’s voice and collaborate with SEIU 775 (Washington State), workers from home care and nursing home organizations with leadership experience will talk with panelists about COVID-19, staffing ratios, advocacy, job quality, job safety, career ladders, and advancement, and intrinsic rewards. These workers will relay their experiences and discuss why they took up these jobs, stay in these jobs, and work to improve these jobs through union strategies. Panelists will ask questions related to agenda setting and co-designing research and policy solutions that simultaneously uplift and align recipient and caregiver voices.

